# Indecision

**Published:** 1878-12

**Authors:** 


### *INDECISION.*

To be constantly changing one's plans and views; to enter enthusiastically, perhaps wildly, into a project to-day, which is to be abandoned to-morrow, and forgotten the next day, is evidence of a weak mind, though the person may possess a thousand graceful accomplishments, or the knowledge of many books. Time and opportunities for usefulness are frittered away, and life becomes indeed a "dream," and nothing more. The eyes may turn sadly back over the "desert" of life, that steadily enlarges its barren boundaries with the waning years, but habits of indolence and of vacillation, early formed, are usually fixed; and a late determination to live to some purpose is soon forgotten, and the unhappy being "resolves and re-resolves and dies the same." The most important practical lesson a child can be taught is—*always finish worthily whatever is worthy to be undertaken.*

